# Blood donors’ knowledge and attitude towards blood donation at North Gondar district blood bank, Northwest Ethiopia: a cross-sectional study

**DOI:** 10.1186/s13104-019-4776-0

**Published:** 2019-11-06

**Authors:** Bamlaku Enawgaw, Aregawi Yalew, Elias Shiferaw

**Affiliations:** Department of Hematology& Immunohematology, School of Biomedical and Laboratory Sciences, College of Medicine and Health Sciences, University of Gondar, P.O. Box 196, Gondar, Ethiopia

**Keywords:** Blood donation, Blood donors, Knowledge, Attitude, Gondar, Northwest Ethiopia

## Abstract

**Objective:**

Blood transfusion saves millions of lives. But, the need and the actual number of donations are not balanced in Ethiopia. The actual reason is not clearly assessed; however, level of knowledge and attitude may be the main contributing factors. Thus, the current study aimed to assess blood donors’ knowledge and attitude towards blood donation at North Gondar district blood bank.

**Results:**

Of 401 blood donors, 142 (35.4%) and 379 (94.5%) were had adequate knowledge and positive attitude towards blood donation, respectively. About 343 (85.5%) of study participants had no previous experience of blood donation. Perceptions of fear of pain, medically unfitness to donate and lack of information on when, where and how to donate blood were mentioned as a reason for not donating blood. Educational status and residence were significantly associated with knowledge of blood donors. On the other hand, participants with secondary and higher education were more likely to have good attitude towards blood donation. Thus, blood banks should design strategies for health education about blood donation and transfusion.

## Introduction

Blood donation is remained the major source of blood and blood components worldwide. Even though extensive promising research have come up, a true substitute for blood and blood components is not available [1]. Donated blood is an essential component in the management of many diseases. It is the main lifesaving for an individual with loss of large volumes of blood from accidents, hemorrhages or surgery [2].

The source for blood to be transfused relies mainly on voluntary non-remunerated blood donors [3]. Even though over a million of blood units are collected every year, many more millions still need to be collected to meet the global demand, ensure the sufficient and timely provision of blood [4]. However, the demand and supply are not being balanced; the demand is escalating. This is the reason why in Sub-Saharan Africa replacement and paid donors are common in contrary to voluntary and non-remunerated donors [5].

Evidences showed that the annual global blood collection is 112.5 million units of blood. Over half of these units of blood are collected in developed countries. The blood donation rate per 1000 people in high income countries is more than fivefold compered to low income countries (33.1 vs 4.6 donations). Voluntary blood donors cover over 90% of donations in developed countries while they account below 50% in developing countries [6].

Ethiopia is a country with high maternal mortality (676 per 100,000) and high motor accident and with a large nonimmune population for malaria [7]. There is insufficiency and in-equitability in access to blood. The average annual national requirement for blood in Ethiopia is 100,000 units per year, but only 43% is collected [8]. From WHO African countries, Ethiopia has the least number of voluntary blood donors (VBD) with 22% which is extremely very low [9].

The availability and safety of blood still remain inadequate to meet the increased demand of blood and blood components particularly in Sub-Saharan Africa like Ethiopia [5, 10]. As a result, these countries try to compensate their blood demand from family replacement or paid donors. But in this type of donors, higher rates of transfusion-transmitted infections have been documented [6]. It is explained that healthy VBD donate their blood by their own free will without any pressure, whereas family replacement donors donate blood for fear of loos of their relatives without considering their health status [11].

The actual reason why large proportion of the potentially eligible population do not actively donate blood is not clearly assessed in Ethiopia. The blood donors’ attitude, beliefs, and knowledge may be a factor for not being a blood donor. Thus, the current study was aimed to assess blood donors’ knowledge and attitude towards blood donation at North Gondar blood bank district, Northwest Ethiopia. The findings will be used as a baseline information for the blood banks to plan an effective strategy to increase and maintain safe and adequate blood supply.

## Main text

### Methods

#### Study setting and population

A cross-sectional study was conducted on 401 blood donors at North Gondar blood bank district, Northwest Ethiopia. This blood bank is the only blood bank center located in Gondar for North Gondar, Amhara regional state, at 738 km far from Addis Ababa, capital city of Ethiopia. The blood bank gives serves for the surrounding hospitals in the district.

#### Sample size determination and sampling technique

To determine the required sample size for study, a single population proportion formula was used as denoted below.

$${\text{n}} = \frac{{Z\upalpha/2^{2 } x p\left( {1 - p} \right)}}{{d^{2} }} = \frac{{(1.96)^{2} x \;0.5\left( {1 - 0.5} \right)}}{{(0.05)^{2} }} = 384,$$

where z α/2 = 1.96 at 95% confidence interval, p = 50% because there is no previous study, d = 5% which is tolerable error between the sample and true population.

Considering 5% non-response rate (384 × 5% = 19), the final sample size becomes 403. The study participants were selected randomly from the blood donors in the blood bank.

#### Data collection

The study participants were interviewed during blood donation after obtaining written informed consent. We used a structured pretested questionnaire to collect socio-demographic data, knowledge, attitude, previous blood donation history and reasons for not donating blood previously. In addition to pretest, training was given for data collectors about data collection procedures and objectives of the study. Consistency of the collected data was also checked daily.

#### Knowledge assessment towards blood donation

We used nine questions to assess knowledge of blood donors. For the “correct” and “incorrect” response, “1” and “zero” score were used, respectively. Then the total score was obtained by summing up of the nine knowledge questions score. The scoring ranges from 0 to 9. Those blood donors who answer “five” and more questions correctly from 9 (> 50%) were considered as knowledgeable.

#### Attitude assessment towards blood donation

In this study, attitude was assessed using eight questions. Similar to knowledge scoring “1” and “zero” were used for favorable and unfavorable attitude, respectively. The total score was calculated up to determine the total attitude score. The score was ranged from 0 to 8. Attitude score of half and more (50%) was considered as favorable attitude.

#### Data analysis and interpretation

Data were entered with Epi info 3.5.1 and transported to SPSS 20 for analysis. Descriptive results were summarized and presented with tables. The association of the independent variable with the categorical outcome variable was measured by calculating odds ratio with 95% confidence interval using bivariate and multivariate logistic regression. P value < 0.05 was considered as statistically significant.

### Results

#### Sociodemographic characteristics of study participants

In this study a total of 401 (259 male and 142 female) study participants was included. The response rate was 99.5% (401/403). The mean age of study participants was 26.2 ± 8.2 years ranging from 18 to 57 years old. The majority 212 (52.9%) of them was in the age group of 18–23 years. More than half 235 (58.6%) of the donors had been attending higher education. Majority 188 (46.9%) and 281 (70.1%) of the study participants were students and single in marital status, respectively (Table 1).

**Table 1:** Characteristics of blood donors at North Gondar District Blood Bank, Northwest Ethiopia

Characteristics of blood donors	Frequency	Percentage
Age (years)		
18–23	212	52.9
24–30	97	24.2
> 30	92	22.9
Gender		
Male	259	64.6
Female	142	35.4
Residence		
Rural	128	31.9
Urban	273	68.1
Marital status		
Single	281	70.1
Married	120	29.9
Education		
Up to secondary school attended	166	41.4
Higher education attended	235	58.6
Occupation		
Student	188	46.9
Private work	164	40.9
Government Employee	49	12.2
Previous donation		
Yes	58	14.5
No	343	85.5
Type of donation		
Volunteer	172	42.9
Replacement	229	57.1

#### Knowledge of study participants

From the total study participants, 142 (35.4%) had adequate knowledge towards blood donation. The mean knowledge score of the participants was 4.03 ± 1.44. All of the study participants argued that the importance of blood donation is to save life. From the total study participants, 380 (94.8%) of them had information regarding screening of donated blood for infectious disease before transfusion. But only 20 (5.0%) of the study participants knew HIV, hepatitis virus and syphilis are considered as transfusion transmittable infections (Additional file 1).

#### Attitude of the study participants

Nearly all [379 (94.5%)] of the study participants had favorable attitude towards blood donation. The mean attitude score of the participants was 7.48 ± 1.23. Majority 365 (91.0%) of the participants had a plan to donate blood voluntarily in the future and about 360 (89.8%) of the study participants had plan to become a regular blood donor. Majority 373 (93%) of the study participants had a perception of donation is not harmful to donors (Additional file 1).

#### Previous practice of blood donation

Less than one quarter 58 (14.5%) of study participants had previous history of donation and more than half 229 (57.1%) of them were replacement type of donors. Several factors have been mentioned as a reason for not donating blood previously. About 139 (40.5%) of the blood donors mentioned lack of information (when, where and how to donate) as the main reason for not donating blood previously. Fear of pain, perceptions of unfitting to donate and consideration of donation as harmful practice had also been mentioned as a reason for not donating blood previously (Additional file 2).

#### Factor associated with knowledge of blood donors

In multivariate logistic regression analysis educational status, residence, previous donation history and donor type were significantly associated. Study participants who attained higher education (AOR = 2.8, 95% CI 1.35, 6) and those who lived in urban (AOR = 2.5, 95% CI 1.26, 4.81), history of previous donation (AOR = 2.2, 95%CI 1.13, 4.48) and being volunteer blood donors (AOR = 3.1, 95%CI 1.5, 6.56) were more likely to have adequate knowledge. Age, gender, marital status and occupation were not showed a statistically significant association (Table 2).

**Table 2:** Logistic regression of knowledge with socio-demographic status of the study participants in North Gondar District blood bank, North West Ethiopia

Knowledge assessment items	Knowledge status		COR (95% CI)	AOR (95% CI)
	Adequate	Inadequate		
Age (years)				
18–23	101 (47.6%)	111 (52.4%)	4.3 (2.37, 7.9)	0.76 (0.24, 2.4)
24–30	25 (25.8%)	72 (74.2%)	1.6 (0.82, 3.34)	0.99 (0.36, 2.75)
> 30	16 (17.4%)	76 (82.6%)	1	1
Gender				
Male	69 (26.6%)	190 (73.4)	1	1
Female	73 (51.4%)	69 (48.6)	2.9 (1.9, 4.48)	1.2 (0.68, 2.07)
Residence				
Urban	126 (46.2%)	147 (53.8%)	6 (3.38, 10.67)	2.5 (1.26, 4.81) *
Rural	16 (12.5%)	112 (87.5%)	1	1
Marital status				
Single	126 (44.8%)	155 (55.2%)	5.3 (3.0, 9.4)	1.7 (0.64, 4.66)
Married	16 (13.3%)	104 (86.7%)	1	1
Education				
Up to secondary school attended	15 (9.0%)	151 (91.0%)	1	1
Higher education attended	127 (54.0%)	108 (46.0%)	11.8 (6.57, 21.34)	2.8 (1.35, 6.0) *
Occupation				
Student	103 (54.8%)	85 (45.2%)	1	1
Private work	18 (11.0%)	146 (89.0%)	0.1 (0.06, 0.18)	0.5 (0.21, 1.40)
Government employed	21 (42.9%)	28 (57.1%)	0.6 (0.33, 1.17)	1.4 (0.54, 3.78)
Previous donation				
Yes	35 (60.3%)	23 (39.7%)	3.4 (1.89, 5.96)	2.2 (1.13, 4.48) *
No	107 (31.2%)	236 (68.8%)	1	1
Donor type				
Volunteer	105 (61.0%)	67 (39.0%)	8.1 (5.1, 13.0)	3.1 (1.5, 6.56) *
Replacement	37 (16.2%)	192 (83.8%)	1	1

*AOR* Adjusted Odds Ratio, *CI* Confidence interval, *COR* Crude Odds Ratio

*Significant variable with a *p* value less than 0.05 in multivariate analysis

#### Factor associated with attitude of blood donors

Bivariate logistic regression analysis showed that age, educational status, occupation, residence and marital status were significantly associated with attitude of participants. While in multivariate logistic regression analysis none of them were statistically significant. Variables such as gender, previous donation history and donor type did not fulfil the criteria for logistic regression analysis and were excluded from analysis (Table 3).

**Table 3:** Logistic regression of attitude with socio-demographic status of the study participants in North Gondar District blood bank, North west Ethiopia

Attitude assessment items	Attitude status		COR (95%CI)	AOR (95%CI)
	Good	Poor		
Age (years)				
18–23	206 (97.2%)	6 (2.8%)	4.7 (1.67, 13.03)	0.9 (0.17, 5.11)
24–30	92 (94.8%)	5 (5.2%)	2.5 (0.83, 7.5)	1.2 (0.32, 4.25)
> 30	81 (88.0%)	11 (12.0%)	1	1
Gender				
Male	238 (91.9%)	21 (8.1%)	–	–
Female	141 (99.3%)	1 (0.7%)	–	–
Residence				
Urban	267 (97.8%)	6 (2.2%)	6.4 (2.43, 16.67)	2.2 (0.6, 8.21)
Rural	112 (87.5%)	16 (12.5%)	1	1
Marital status				
Single	274 (97.5%)	7 (2.5)	5.6 (2.22, 14.1)	2.2 (0.46, 10.66)
Married	105 (87.5%)	15 (12.5)	1	1
Education				
Have no formal education	38 (76.0%)	12 (14.0%)	1	1
Have formal education	341 (97.2%)	10 (2.8%)	10.8 (4.36, 26.58)	3.4 (0.98, 12.02)
Occupation				
Student/employee	312 (97.5%)	8 (2.5%)	8.1 (3.29, 20.2)	1.6 (0.35, 6.99)
Farmer	67 (82.7%)	14 (17.3%)	1	1
Previous donation				
Yes	57 (98.3%)	1 (1.7%)	–	–
No	322 (93.9%)	21 (6.1%)	–	–
Donor type				
Volunteer	172 (100%)	0	–	–
Replacement	207 (90.4%)	22 (9.6%)	–	–

*NB* Gender, previous donation and donor type do not fulfil the criteria for logistic analysis*, AOR* Adjusted Odds Ratio, *CI* Confidence interval, *COR* Crude Odds Ratio

### Discussion

In this study about one-third of blood donors had adequate knowledge towards blood donation. The result was slightly higher than a study conducted in Jordan which reported that 28.6% of them had adequate knowledge towards blood donation [4]. The possible reason for this discrepancy might type of blood donors. In our study, the number of replacement type of blood donors was relatively low (229 vs 348). It is strongly believed that volunteer blood donors are more likely to have good knowledge towards blood donation compared to replacement type donors and it is considered as major contributing factor for blood donation. This study showed that 61% of voluntary and 16.2% of replacement blood donors had adequate knowledge.

On the other hand, the level of knowledge in this study was lower than studies from Gondar [12], Bahir Dar [13], Wolita Sodo [14, 15], Tigray [16], Birbir Town [17], Harar [18], Basrah, Iraq [19] and India [20]. The difference may be associated with the type of study subjects included in the studies. The above-mentioned studies include medical and health science students and also health care workers. Thus, it is expected that this group of people have high level of knowledge towards blood donation.

In the current study, all of the participants argued that the importance of blood donation is to save life. But a previous report from Gondar town showed a slight deviation result of 88.3% [21]. Similarly, it was higher than a study conducted in Democratic Republic of Congo which showed that only 183 (44.1%) of the study participants strongly advocates this idea [22]. The difference might be due to variation in study subjects (blood donors vs general population in the community).

In this study, participants who attained higher education and lived in urban were more likely to have adequate knowledge towards blood donation. This is supported by studies in Birbir Town [17] and Harar [18] in which individuals with higher education has high level of knowledge. Similarly, those donors who were donate blood previously and volunteer donors were had adequate knowledge compared to their counterparts. This is true that if someone had experience, he/she has high level of knowledge. Thus, it is not surprise that if the donors with previous history had adequate knowledge.

Regarding to attitude, nearly all of the respondents had a good attitude towards blood donation. The finding was slightly higher as compared to the previous report from Gondar [12, 21], Bahir Dar [13], Wolita Sodo [14, 15], Tigray [16], Birbir Town [17], Harar [18], Basrah, Iraq [19] and India [20]. The difference might be due to variation in study method and subjects since the current study was institutional based study conducted among the blood donors.

We tried to assess the association of blood donors’ characteristics with their attitude. Variables such as age, educational status, occupation, residence and marital status were assessed, but none of them showed statistically significant association. Nearly all (94.5%) of the study participants had favorable attitude towards blood donation.

### Conclusion

In this study, attitude towards blood donation was high, but the level of knowledge was inadequate. Education, residence, previous blood donation and donor type were statistically associated with adequate knowledge. To increase the level of knowledge towards blood donation, health education to the community is recommended.

## Limitations

The findings in this study are from one district and only interview-based data were collected. There was no focus group discussion for further analysis of the knowledge and attitude of the participants.

## Supplementary information

**Additional file 1.** Knowledge and attitude questions response of blood donors towards blood donation at North Gondar District Blood Bank, Northwest Ethiopia.

**Additional file 2.** Blood donation practice of blood donors North Gondar District Blood Bank, Northwest Ethiopia.

## Data Availability

The datasets used and/or analyzed during the current study available from the corresponding author on reasonable request.

## Abbreviations

AOR: Adjusted Odds Ratio
CI: confidence interval
COR: Crude Odds Ratio
HBV: hepatitis B virus
HCV: hepatitis C virus
VBD: voluntary blood donors
WHO: World Health Organization
VNRBD: voluntary non-remunerated blood donors
