# Supplementary material for: Blood donors’ knowledge and attitude towards blood donation at North Gondar district blood bank, Northwest Ethiopia: a cross-sectional study
Source: BMC Res Notes. 2019 Nov 6;12:729. doi: 10.1186/s13104-019-4776-0 (PMC6836355; doi:10.1186/s13104-019-4776-0)
Supplement: Supplementary file 1 — Additional file 1. Knowledge and attitude questions response of blood donors towards blood donation at North Gondar District Blood Bank, Northwest Ethiopia. [file 13104_2019_4776_MOESM1_ESM.docx]

**Additional file 1:** Knowledge and attitude questions response of blood donors towards blood donation at North Gondar District Blood Bank, Northwest Ethiopia

| **Knowledge assessment items** | **Response** | |
| --- | --- | --- |
|  | **Correct response**  **N (%)** | **Incorrect response**  **N (%))** |
| Knowledge about place of blood donation | 88 (21.9%) | 313 (78.1%) |
| Importance of blood donation | 401 (100.0%) | 0 |
| Minimum age eligible for blood donation | 135 (33.7%) | 266 (66.3%) |
| Maximum age eligible for blood donation | 44 (11.0%) | 357 (89.0%) |
| Minimum weight eligible for blood donation | 288 (71.8%) | 113 (28.2%) |
| Minimum time interval for blood donation | 281 (70.1%) | 120 (29.9%) |
| Knowledge about TTIs | 20 (5.0%) | 381 (95.0%) |
| Does donated blood will undergo screening? | 380 (94.8%) | 21 (5.2%) |
| Is there a possibility of recipients to be exposed to infection/ harm during transfusion? | 8 (2.0%) | 393 (98.0%) |
| **Attitude assessment items** | **Favorable response**  **N (%)** | **Unfavorable response**  **N (%)** |
| Do you think donation is harm full to donors | 373 (93.0%) | 28 (7.0%) |
| Will you donate voluntarily | 365 (91.0%) | 36 (9.0%) |
| Will you donate to unknown person if you are asked | 358 (89.3%) | 43 (10.7%) |
| Will you ask for a monetary compensation for blood donation | 376 (93.8%) | 25 (6.2%) |
| Will you discuss with your friends and your family about blood donation | 383 (95.5%) | 18 (4.3%) |
| Will you encourage others for donation | 387 (96.5%) | 14 (3.5%) |
| Do you have a plan to become a regular blood donor | 360 (89.8%) | 41 (10.2%) |
| Will you tell your true health status to doctors before donation? | 399 (96.5%) | 2 (0.5%) |
