# Supplementary material for: Blood donors’ knowledge and attitude towards blood donation at North Gondar district blood bank, Northwest Ethiopia: a cross-sectional study
Source: BMC Res Notes. 2019 Nov 6;12:729. doi: 10.1186/s13104-019-4776-0 (PMC6836355; doi:10.1186/s13104-019-4776-0)
Supplement: Supplementary file 2 — Additional file 2. Blood donation practice of blood donors North Gondar District Blood Bank, Northwest Ethiopia. [file 13104_2019_4776_MOESM2_ESM.docx]

**Additional file 2:** Blood donation practice of blood donors North Gondar District Blood Bank, Northwest Ethiopia

| **Blood donation practice** | | **Frequency** | **Percentage** |
| --- | --- | --- | --- |
| Previous history of donation | Yes | 58 | 14.5 % |
|  | No | 343 | 85.5 % |
| Type of donation | Volunteer | 172 | 42.9 % |
|  | Replacement | 229 | 57.1 % |
| Number of donations | For the first time | 26 | 45 % |
|  | 2- 3 times | 26 | 45 % |
|  | > 3 times | 6 | 10 % |
| Reason for not donating blood previously | Perceptions of am not medically fit | 60 | 15 % |
|  | Lack of information on when, where and how to donate | 139 | 34.7 % |
|  | Fear of pain | 62 | 15.5 % |
|  | Influence from friends/ families | 31 | 7.7 % |
|  | Don’t like the idea of donation by itself | 27 | 6.7 % |
|  | Other reasons | 16 | 4.2 % |

***Other reasons include****: - am not in the age to donate, carelessness and lack of concern, lack of opportunity to donate*
